# Comparison of Bovine- and Porcine-Derived Decellularized Biomaterials: Promising Platforms for Tissue Engineering Applications

**DOI:** 10.3390/pharmaceutics15071906

**Published:** 2023-07-08

**Authors:** Hussein M. El-Husseiny, Eman A. Mady, Masahiro Kaneda, Kazumi Shimada, Yasumoto Nakazawa, Tatsuya Usui, Mohamed Elbadawy, Yusuke Ishihara, Moeko Hirose, Yohei Kamei, Ahmed S. Doghish, Hesham A. El-Mahdy, Walaa A. El-Dakroury, Ryou Tanaka

**Affiliations:** 1 Laboratory of Veterinary Surgery, Department of Veterinary Medicine, Faculty of Agriculture, Tokyo University of Agriculture and Technology, 3-5-8 Saiwai Cho, Fuchu-shi 183-8509, Tokyo, Japan; kazumi-s@go.tuat.ac.jp; 2 Department of Surgery, Anesthesiology and Radiology, Faculty of Veterinary Medicine, Benha University, Moshtohor, Toukh 13736, Elqaliobiya, Egypt; 3 Laboratory of Veterinary Physiology, Department of Veterinary Medicine, Faculty of Agriculture, Tokyo University of Agriculture and Technology, 3-5-8 Saiwai Cho, Fuchu-shi 183-8509, Tokyo, Japan; s211566z@st.go.tuat.ac.jp; 4 Department of Animal Hygiene, Behavior, and Management, Faculty of Veterinary Medicine, Benha University, Moshtohor, Toukh 13736, Elqaliobiya, Egypt; 5 Laboratory of Veterinary Anatomy, Division of Animal Life Sciences, Faculty of Agriculture, Tokyo University of Agriculture and Technology, 3-5-8 Saiwai Cho, Fuchu-shi 183-8509, Tokyo, Japan; kanedam@cc.tuat.ac.jp; 6 Department of Biotechnology and Life Science, Tokyo University of Agriculture and Technology, Koganei 184-8588, Tokyo, Japan; yasumoto@cc.tuat.ac.jp (Y.N.); s221510z@st.go.tuat.ac.jp (M.H.); s233892x@st.go.tuat.ac.jp (Y.K.); 7 Laboratory of Veterinary Pharmacology, Department of Veterinary Medicine, Faculty of Agriculture, Tokyo University of Agriculture and Technology, 3-5-8 Saiwai Cho, Fuchu-shi 183-8509, Tokyo, Japan; fu7085@go.tuat.ac.jp (T.U.); mohamed.elbadawy@fvtm.bu.edu.eg (M.E.); ishiharatat@gmail.com (Y.I.); 8 Department of Pharmacology, Faculty of Veterinary Medicine, Benha University, Moshtohor, Toukh 13736, Elqaliobiya, Egypt; 9 Department of Biochemistry, Faculty of Pharmacy, Badr University in Cairo (BUC), Badr City 11829, Cairo, Egypt; ahmed_doghish@azhar.edu.eg; 10 Department of Biochemistry, and Molecular Biology, Faculty of Pharmacy (Boys), Al-Azhar University, Nasr City 11651, Cairo, Egypt; heshamabbas@azhar.edu.eg; 11 Department of Pharmaceutics and Industrial Pharmacy, Faculty of Pharmacy, Badr University in Cairo, Badr City 11829, Cairo, Egypt; w_dakroury@yahoo.com

**Keywords:** biomaterials, decellularization, bovine pericardium, porcine pericardium, porcine tunica vaginalis, tissue engineering

## Abstract

Animal-derived xenogeneic biomaterials utilized in different surgeries are promising for various applications in tissue engineering. However, tissue decellularization is necessary to attain a bioactive extracellular matrix (ECM) that can be safely transplanted. The main objective of the present study is to assess the structural integrity, biocompatibility, and potential use of various acellular biomaterials for tissue engineering applications. Hence, a bovine pericardium (BP), porcine pericardium (PP), and porcine tunica vaginalis (PTV) were decellularized using a Trypsin, Triton X (TX), and sodium dodecyl sulfate (SDS) (Trypsin + TX + SDS) protocol. The results reveal effective elimination of the cellular antigens with preservation of the ECM integrity confirmed via staining and electron microscopy. The elasticity of the decellularized PP (DPP) was markedly (*p* < 0.0001) increased. The tensile strength of DBP, and DPP was not affected after decellularization. All decellularized tissues were biocompatible with persistent growth of the adipose stem cells over 30 days. The staining confirmed cell adherence either to the peripheries of the materials or within their matrices. Moreover, the in vivo investigation confirmed the biocompatibility and degradability of the decellularized scaffolds. Conclusively, Trypsin + TX + SDS is a successful new protocol for tissue decellularization. Moreover, decellularized pericardia and tunica vaginalis are promising scaffolds for the engineering of different tissues with higher potential for the use of DPP in cardiovascular applications and DBP and DPTV in the reconstruction of higher-stress-bearing abdominal walls.

## 1. Introduction

The current scarcity of human organs and tissues appropriate for life-saving transplants is expected to persist in the future despite the great endeavors to close the donation gap [1,2,3,4,5,6]. Hence, it is highly crucial to introduce efficient alternatives to avoid the high incidence of deaths among patients that occur due to the long waiting lists for donor organs/tissues or due to receiving suboptimal treatments [7,8,9,10,11]. Animal-originated organs/tissues (xenotransplants) have been recently introduced as efficient and potential alternatives to solve this problem. Moreover, a significant advancement has been made in this active field of research [12,13,14,15]. Animal organs and tissues have been employed for decades in the regeneration and engineering of different body tissues [16].

Decellularization is a tissue engineering approach that involves the elimination of cellular and nuclear components while maintaining the extracellular matrix’s (ECM) composition, biological activity, and mechanical integrity [17,18]. This entails increasing cellular material clearance while minimizing ECM loss. Employment of the ECM generated from decellularized tissues is a leading strategy in regenerative medicine [19]. It affects cellular differentiation, mitogenesis, chemotaxis, and tissue-remodeling responses [19,20,21,22,23,24,25]. Decellularization also lessens antigenicity, especially in xenografts, which is a very important feature [26].

A variety of decellularization regimens have been investigated by employing various chemicals, concentrations, and exposure durations [27,28]. Tissue treatment using glutaraldehyde (GA) is common post-decellularization. However, GA-fixed decellularized scaffolds have been reported to provoke high immune and inflammatory reactions and present lower regeneration and tissue engineering capacities. This was confirmed by the high rate of proinflammatory cytokines release and the lower rate of wound-healing-promoting cytokine IL-1Ra release in the GA-treated matrix compared to the untreated control [29].

Decellularized animal tissues are extensively used as scaffolds to repair/regenerate damaged tissues. Their natural collagen and elastin fibers can provide valuable mechanical and non-thrombogenic properties. Bovine pericardium (BP) has been considered the gold-standard source of material for cardiovascular uses [30]. Despite their availability and biocompatibility, BP scaffolds are commonly cross-linked to increase their stability and immune compatibility. However, tissue fixation treatments may increase calcification and limit recipient cellular ingrowth [31,32]. Porcine pericardial (PP) tissue, which is structurally more akin to human tissue, may be a better biomaterial for tissue engineering applications than BP, which has been the subject of numerous studies [33]. For instance, both sources of pericardium have the same collagen structure and organization [30]. However, the PP is thinner, which elevates the effectiveness of the decellularization process in terms of the removal of xenogeneic materials [34]. Tunica vaginalis is the serous tissue that covers the testis and epididymis. It is produced from the peritoneum and mimics the characteristics of the natural abdominal wall. Despite being a biowaste from the meat industry, decellularized tunica vaginalis presented satisfactory physicochemical characteristics promising for applications in tissue engineering [35]. Many pediatric urologists still prefer tunica vaginalis as the substrate of choice for corporoplasty for treating ventral curvature due to its simplicity in harvesting and the absence of donor site morbidity [36].

The ideal scaffold should present a network that enables functional cell–cell and/or cell–ECM interactions. The right topography, microstructure, and mechanical properties of the tissue are crucial for efficient regeneration in vivo [37]. Hence, it is still difficult to develop an ECM that is less immunogenic, well maintained, and has the biological and biomechanical capacity to promote cellular repopulation and enable tissue remodeling. In addition, it is crucial to deeply understand the ECM niche’s ability to control cellular behavior in the scaffold of choice to master its grafting on the damaged site to renew the tissue and restore its function [38]. In the present study, we aimed to develop biocompatible, biodegradable, elastic, and mechanically strong biomaterials which are less immunogenic and support healing processes to be employed for the engineering of various body tissues. We assessed the effects of the decellularization process on the structure, biomechanics, and biocompatibility of BP, porcine pericardium (PP), and porcine tunica vaginalis (PTV). Moreover, we aimed to assess the potential of employing stem-cell-seeded scaffolds as a new regenerative modality for engineering various body tissues.

## 2. Materials and Methods

### 2.1. Decellularization Protocol

**Figure 1:**
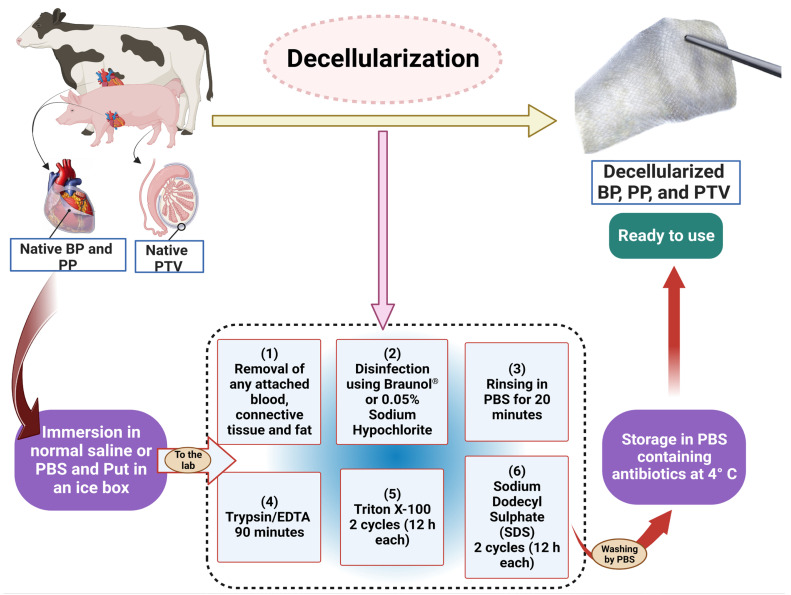
Schematic illustration of the decellularization protocol employed in the present study.

The native tissues were collected from the local slaughterhouse in Tokyo, Japan, from healthy animals. Bovine pericardium (BP) tissues were obtained from cows of both sexes aged 18–32 months. Moreover, the porcine pericardium (PP) was collected from pigs of both sexes and the porcine tunica vaginalis (PTV) was collected from boars. All pigs were about 5–6 months of age at the time of slaughtering. All procedures from collection to the end of decellularization are presented in Figure 1. Tissues were kept in phosphate-buffered saline (PBS) and transferred to the laboratory in ice boxes. In the laboratory, we removed the attached fat and excess connective tissues, washed them well with PBS, and disinfected the tissues using sodium Braunol^®^ (B. Braun) with shaking for 5 min. After that, they were rinsed with PBS for 20 min before beginning the decellularization procedures using the Trypsin (Trypsin/EDTA, T4049-100ML, Sigma Aldrich, St. Louis, MI, USA), Triton X (TX-100, T8787-100ML, Sigma Aldrich, St. Louis, MI, USA), and sodium dodecyl sulfate (SDS, L3771, Sigma Aldrich, St. Louis, MI, USA) (Trypsin + TX + SDS) protocol described in [16]. Firstly, the tissues were incubated in 0.125% Trypsin−0.05% EDTA for 90 min, followed by two cycles of incubation in 0.5% TX for 12 h each. After that, two 12 h cycles of incubation in 0.5% SDS were achieved. Then, the tissues were rinsed twice in water for 12 h/cycle. Eventually, fourteen 12 h cycles of rinsing in PBS were accomplished. All incubation procedures were carried out at room temperature and under 200 rpm shaking using an orbital shaker. The decellularized tissues were preserved in PBS containing antibiotics (penicillin 1 mg/mL, streptomycin 1 mg/mL, amphotericin 1 mg/mL; 161-23181, Fujifilm Wako Pure Chemical Corporation, Osaka, Japan) at 4 °C till the time of use (Figure 1). All procedures were carried out following the Tokyo University of Agriculture and Technology’s Institutional Animal Care and Use Committee’s assessment and approval (Approval No. R05-90).

### 2.2. In Vitro Evaluation of the Decellularization Process

#### 2.2.1. Histology

To assess the efficacy of the decellularization process, H & E staining procedures were conducted to detect the nuclear materials as described by [39]. Briefly, the native and decellularized tissues segments (n = 6/tissue) were fixed in 10% neutral buffered formalin (Sigma-Aldrich, St. Louis, MO, USA) for 1 h, embedded in paraffin, cut into 5 µm sections, and stained with hematoxylin and eosin (H & E, Sigma Aldrich, St. Louis, MO, USA). The same procedures were adopted to check the biocompatibility of the decellularized biomaterials seeded with r-AdMSCs on day 30 post-seeding.

#### 2.2.2. Scanning Electron Microscope (SEM)

The morphology of the native and decellularized materials (n = 3) was assessed using a scanning electron microscope (SEM) (JSM 6510 JEOL Ltd., Tokyo, Japan) [38,40]. Briefly, the specimens were sliced into pieces of about 1 mm^3^, fixed with 2.5% glutaraldehyde, and postfixed in 1% osmium tetroxide. Both were buffered using 0.1 mol/L sodium cacodylate buffer pH 7.2. Then, the tissues were rinsed in the buffer and dehydrated in (15%, 30%, 50%, 70%, 90%, and 100%) serially increasing ethanol concentration for 10 min each before being dried with CO_2_ using the critical point drying technique. Ultimately, the sections were coated with colloidal gold and examined using SEM at 10 KV acceleration voltage.

#### 2.2.3. Assessment of the Weight Loss

The initial (baseline) weight (W0) was measured after vacuum drying of the preserved tissue samples to eliminate the water residues (pre-impregnation). Then, the samples were immersed in PBS and incubated at 37 °C. The PBS solution was serially changed every two days (impregnation). On day 7, the tissues were removed from the PBS, rinsed using distilled water, and completely vacuum-dried (post-impregnation). We measured the weights of these tissue samples again (W1). Subsequently, the following formula was employed to estimate the rate of weight loss (%), where W0 and W1 are the pre- and post-impregnation weights, respectively: weight loss (%) = [(W0 − W1)/W0] × 100 [41].

#### 2.2.4. Biomechanics (Tensiometric Evaluation)

To study the biomechanics of different tested tissues, representative 25 × 5 mm^2^ (length × width) samples from the native and decellularized tissues (n = 6) were used. The thickness of each sample was measured from its center using a digital caliper. For each sample, the edge was clamped at both sides with custom grips and pulled to a length of 15 mm at a crosshead speed of 2 mm/min until the breakage point was attained, and the data were analyzed. The measurements were conducted using a 100 N load cell on a tensile testing machine (EZ-Test, Shimadzu, Kyoto, Japan). Stress–strain curves were gained depending on the measured strength, length, and thickness of the sample. The ultimate tensile strength (MPa), Young’s (elastic) modulus (MPa), and elongation at breakage (%) were measured [42,43]. 

### 2.3. Isolation and Characterization of Rat-Adipose-Derived Mesenchymal Stem Cells (r-ADMSCs)

Inguinal adipose tissue was collected under strict sterile conditions. The isolation of inguinal r-AdMSCs was cultured and characterized for pluripotency, stemness, and multilineage differentiation using flowcytometry, qPCR, and RT-PCR in our recently published study [22].

### 2.4. In Vitro Assessment of the Cytocompatibility

The cytocompatibility of the decellularized BP, PP, and PTV tissues was assessed in vitro according to a method modified after [38]. Briefly, 1 × 3 mm^2^ decellularized tissues (n = 3/each tissue) were incubated for 24 h in an antibiotic mixture solution comprising 1 mg of penicillin + 1 mg of streptomycin + 0.5 mg of amphotericin dissolved in PBS. Then, they were put in DMEM at 4 °C overnight. After that, they were cultured in Terasaki 3D culture plate, cultivated with r-AdMSCs (n = 5 rats, 5 × 10^4^ cells/decellularized tissue sample), and incubated in DMEM for 24 h at 37 °C and 5% CO_2_. The tissue specimens were then placed in a nonadherent 6-well plate, supplemented with DMEM, and incubated again. The medium was replaced every 2–3 days. The tissues were extracted on days 1, 7, 14, and 30 of the culturing and fixed using 4% PFA for 10 min. Samples embedded in paraffin were stained using hematoxylin and eosin (H & E).

### 2.5. In Vivo Assessment of Biocompatibility

The in vivo evaluation was performed via subcutaneous implantation of different biomaterials in a rat model. The tissue specimens were cut into 10 × 10 mm^2^ pieces. Thirty healthy male SD rats (2 months of age, and 250–300 g weight) were assigned into six different groups (NBP, NPP, NPTV, DBP, DPP, DPTV; 5 rats/group). Animals were anesthetized using an MMB intraperitoneal injection protocol (medetomidine 0.15 mg/kg, midazolam 2 mg/kg, butorphanol 2.5 mg/kg). Following shaving of the hair and sterilization of the skin, a roughly 2 cm skin incision was performed in each group for the implantation of the biomaterials subcutaneously at the back region. Then, the incisions were sutured using 3.0 silk suture material [44]. To prevent infection after the closure of the skin incision, gentamicin was injected intraperitoneally for 3 successive days. Moreover, carprofen (5 mg/kg subcutaneous) was administered pre-surgery and post-surgery for 2 days to prevent animal pain after surgery. After awakening. Rats were placed in a recovery cage with the temperature maintained at 37 °C overnight and then housed in cages in a room at 20–25 °C and 40–60% humidity, with a 12 h light–dark cycle. One month after implantation, animals were euthanized using an overdose of isoflurane inhalation according to [45]. Then, the collected implants were assessed using H & E stain. Infiltrated cells and newly formed vessels were quantified for each sample in five microscopic fields at 400X magnification power [44]. All procedures were carried out following the Tokyo University of Agriculture and Technology’s Institutional Animal Care and Use Committee’s assessment and approval (Approval No. R05-90).

### 2.6. Statistical Analysis

Comparison between various tissues was accomplished via one-way analysis of variance (ANOVA) followed by Tukey’s post hoc test. GraphPad Prism software version 9 (GraphPad Software, Inc., La Jolla, CA, USA) was used. Data were expressed as mean ± standard deviation (SD). *p* < 0.05 was labeled as statistically significant.

## 3. Results

### 3.1. Histological Assessment

**Figure 2:**
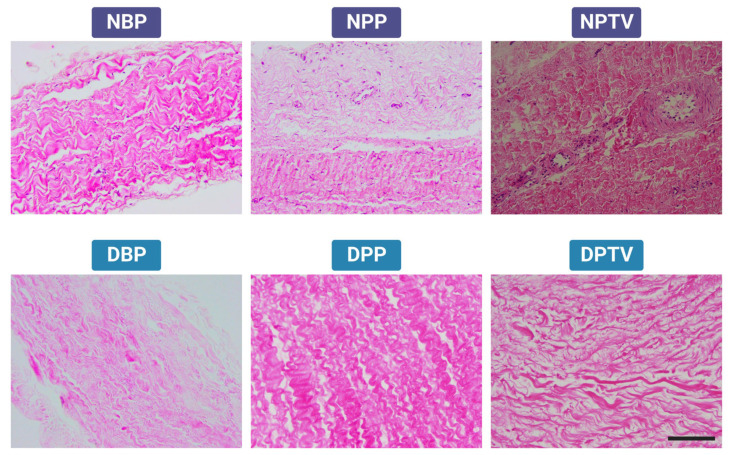
Histological (H & E staining) evaluation of different biomaterials before and after decellularization. NBP, native bovine pericardium; NPP, native porcine pericardium; NPTV, native porcine tunica vaginalis. Scale bar: 200 µm.

The H & E-stained tissues revealed the presence of many nuclei in the native pericardia and tunica vaginalis tissues. The decellularization process could efficiently remove the cellular part of the decellularized tissues with the preservation of intact ECMs. The wavy appearance of the collagen bundles characteristic of the native tissues, NBP, NPP, and NPTV, was observed in the decellularized tissues, DBP, DPP, and DPTV (Figure 2).

### 3.2. Scanning Electron Microscopy

**Figure 3:**
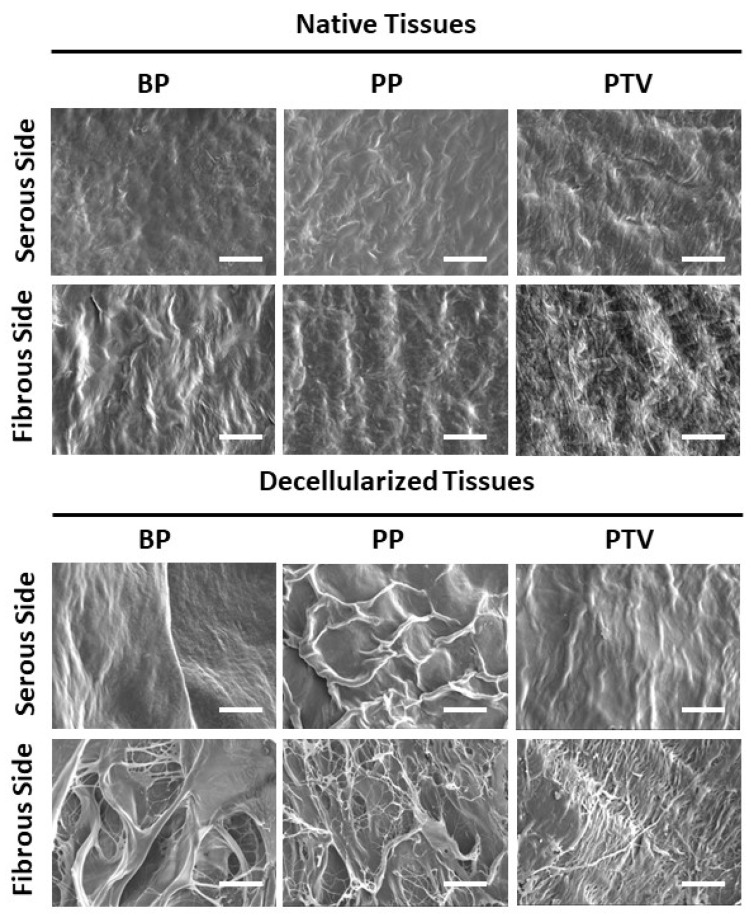
Scanning electron microscopy of the native and decellularized BP, PP, and PTV assessed on both the serous and fibrous sides of the materials. Scale bar: 200 µm.

To visualize more structural details, SEM was performed. The serous side was smoother, with better-arranged collagen fibers. The serous surface of the native BP, PP, and PTV appeared more regular due to the presence of the mesothelial cell layer, which was removed in the decellularized BP, PP, and PTV. Hence, the serous side in the decellularized biomaterials appeared wavier due to the sub-mesothelial collagen tissue (Figure 3). On the fibrous side, the collagen bundles appeared more organized in the native tissues. After decellularization, the collagen fibers appeared looser and wider on the fibrous surface of the decellularized tissues. Moreover, they were looser in the NBP and DBP tissues than in the other tissues (Figure 3).

### 3.3. Weight Loss (%)

**Figure 4:**
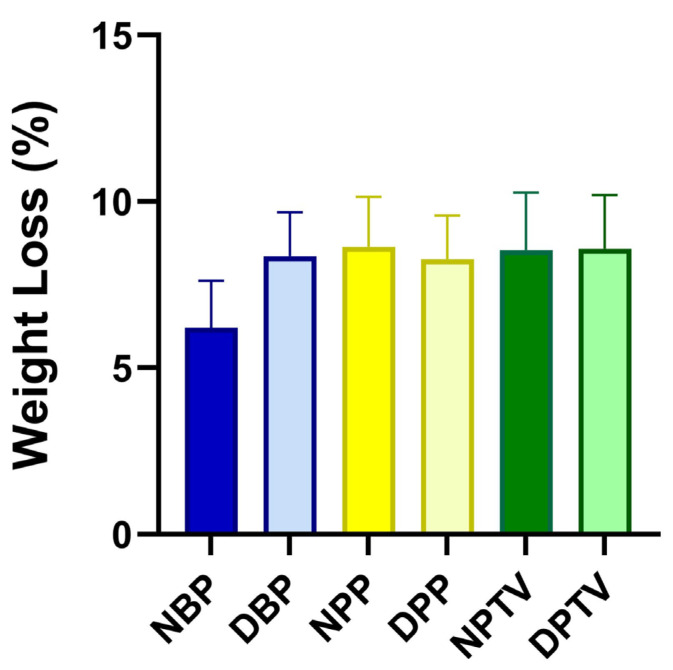
Assessment of the weight loss % in various biomaterials following 7 days of impregnation in PBS.

The findings of the in vitro weight loss reveal that there was no significant change in the weight loss either between the different native tissues (6.21 ± 1.99, 8.65 ± 2.12, and 8.54 ± 2.45 for NBP, NPP, and NPTV; respectively) or between the decellularized tissues (8.36 ± 1.86, 8.27 ± 1.86, and 8.58 ± 2.29 for DBP, DPP, and DPTV; respectively (Figure 4). In addition, the comparison of the weight loss of each material before and after the decellularization showed no marked alterations (Figure 4).

### 3.4. Biomechanical Characters

**Figure 5:**
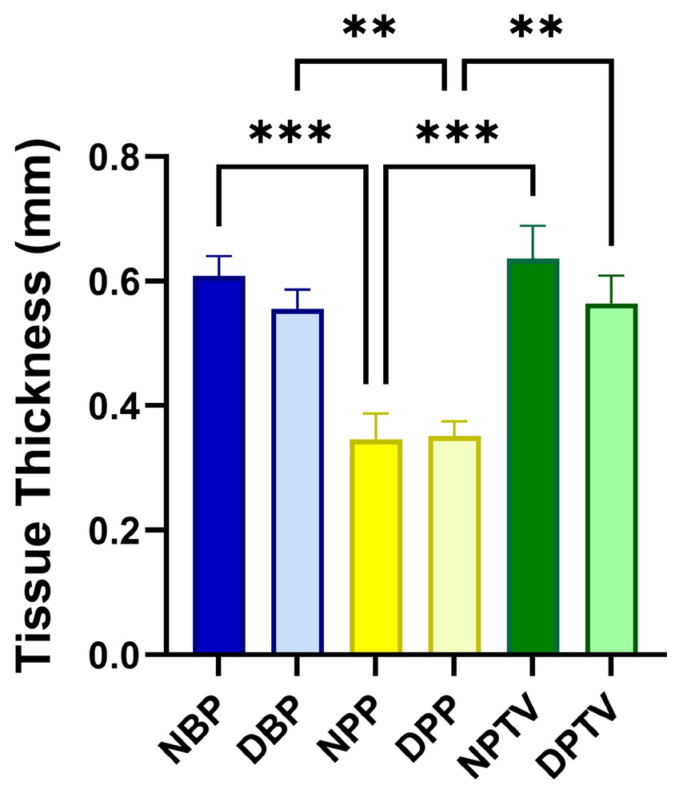
Thickness of various biomaterials employed in the present study before and after decellularization. ** *p* < 0.01, and *** *p* < 0.001.

**Figure 6:**
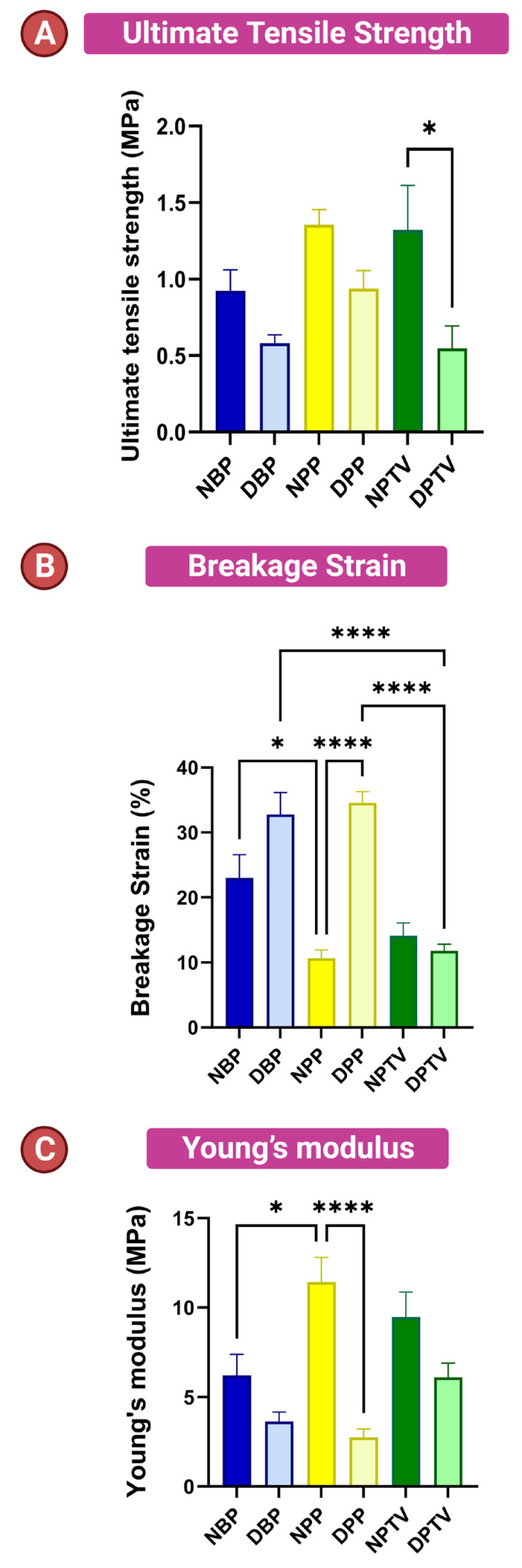
Biomechanical characters of native and decellularized BP, PP, and PTV. Ultimate tensile strength (**A**), breakage strain (**B**), and Young’s modulus (**C**) Comparison of the biomechanical properties between the native and decellularized materials. * *p* < 0.05, and **** *p* < 0.0001.

The thickness of different tissues in this study is shown in Figure 5. The thickness of NBP and NPTV was significantly (*p* < 0.001) higher than that of the NPP. Additionally, the thickness of DBP and DPTV was markedly (*p* < 0.001) elevated compared to that of the DPP (Figure 5). On the contrary, a comparison between the scaffolds revealed that there was no significant change in the thickness of various biomaterials before and after the decellularization (0.61 ± 0.07, 0.56 ± 0.07, 0.34 ± 0.09, 0.35 ± 0.05, 0.64 ± 0.12, 0.56 ± 0.9 for NBP, DBP, NPP, DPP, NPTV, and DPTV; respectively) (Figure 5). A biomechanics evaluation revealed that for the ultimate tensile strength, there was no significant change between the native tissues and the decellularized tissues (Figure 6A). Moreover, the decellularization did not affect the tensile strength of the materials except in the tunica vaginalis, where there was a significant (*p* < 0.05) decrease in the UTS of the DPTV compared to the NPTV (Figure 6A). The breakage strain was significantly higher in the NBP versus the NPP (*p* < 0.05), DBP versus DPTV (*p* < 0.0001) and DPP versus DPTV (*p* < 0.0001) (Figure 6 B). The decellularization significantly affected the breakage strain of the porcine pericardium as there was a marked (*p* < 0.0001) increase in the strain (%) of the DPP compared to the NPP (Figure 6B). The Young’s modulus was significantly (*p* < 0.05) elevated in the NPP compared to the NBP (Figure 6C). The Young’s modulus of the bovine pericardium and porcine tunica vaginalis did not change significantly after the decellularization. However, it was significantly (*p* < 0.0001) reduced in the DPP compared to the NPP (Figure 6C).

### 3.5. In Vitro Cytocompatibility

**Figure 7:**
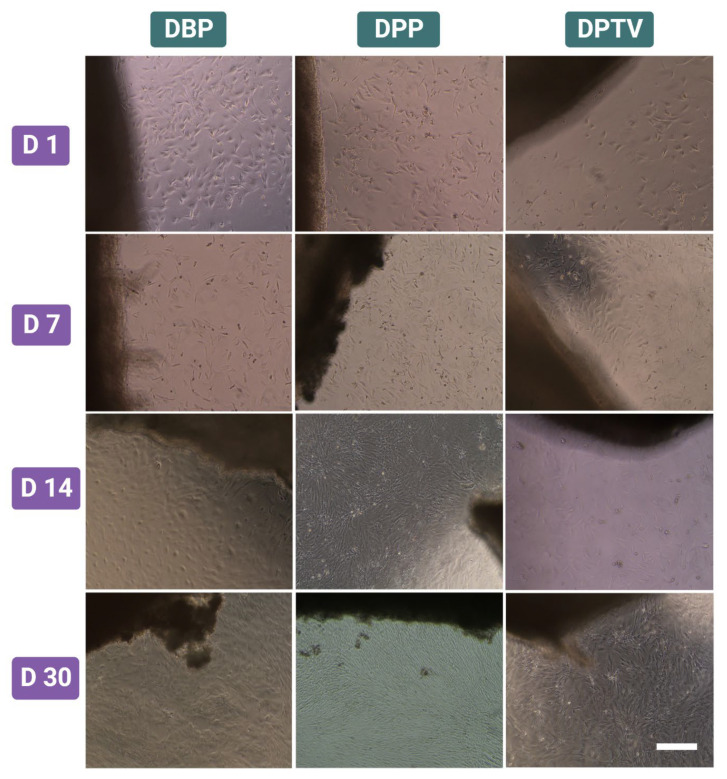
Cytocompatibility and bioactivity of decellularized materials assessed at different time points (days 1, 7, 14, and 30) of the study. Scale bar: 200 µm.

**Figure 8:**
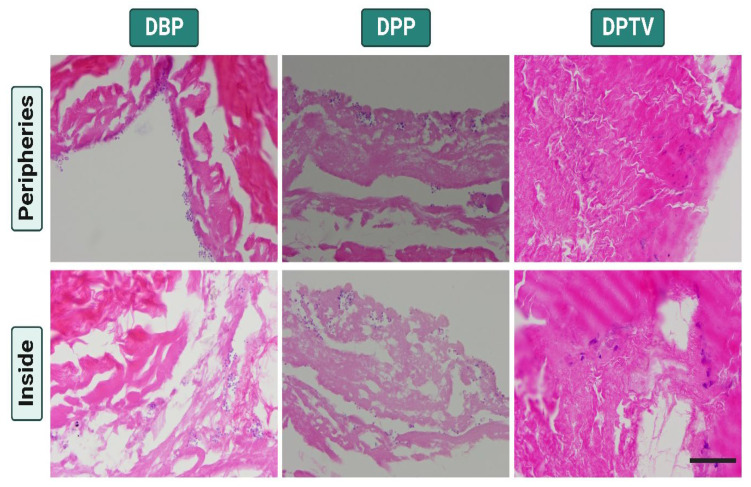
Hematoxylin and eosin (H & E) staining of the r-AdMSC-seeded materials evaluated after 30 days of culturing. Adherent stem cells were observed on the peripheries of the biomaterials and within their matrices. Scale bar: 200 µm.

To assess the biocompatibility of the various tested decellularized biomaterials, we cultured the scaffolds with r-AdMSCs for 30 days. The cultured cells presented obvious growth over time (Figure 7). The DBP and DPP showed higher cell proliferation compared to the DPTV. On day 30, the cells harvested on the DPP exhibited superior growth. To confirm the cell adherence to the surface of the decellularized biomaterials, H & E staining was performed, which revealed the presence of the AdMSCs on the peripheries of the decellularized matrices as well as inside their matrices. These findings were more obvious in DBP and DPP scaffolds and less obvious in DPTV. This indicates that both DBP and DPP have higher biocompatibility than DPTV (Figure 8).

### 3.6. In Vivo Assessment of Biocompatibility

**Figure 9:**
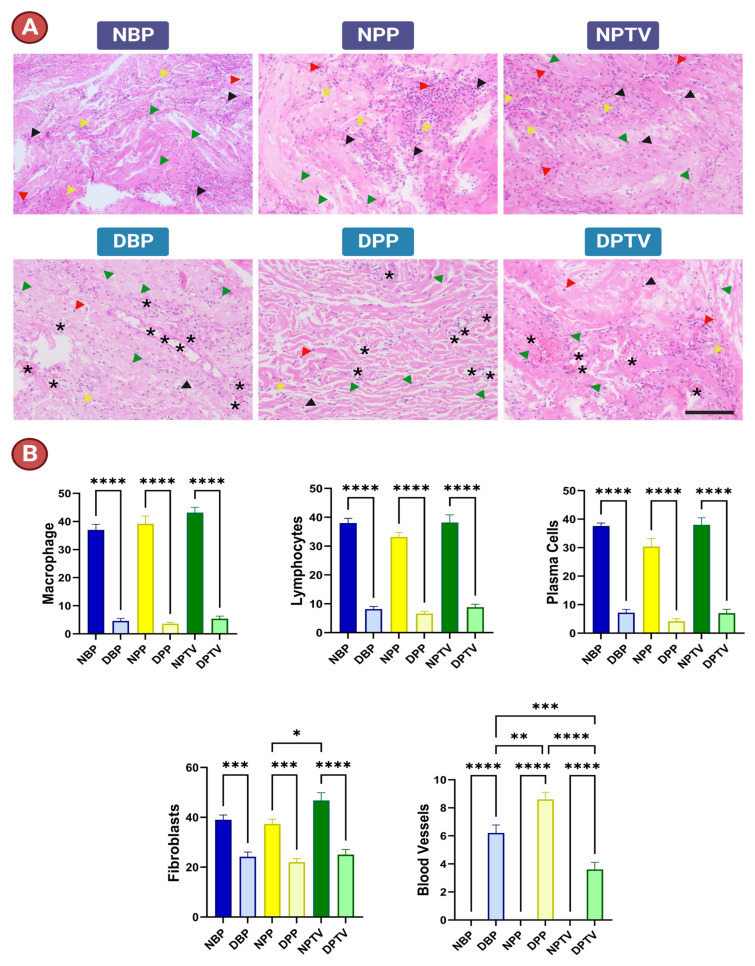
In vivo assessment of the biocompatibility of different tissues in the present study. Histological assessment of different implanted materials (**A**). Quantification of the infiltrating inflammatory cells and the newly formed blood vessels (**B**). Red arrow heads, macrophages; black arrow heads, lymphocytes; yellow arrow heads, plasma cells; green arrow heads, fibroblasts; and black asterisk, blood vessels. Scale bar: 200 µm. * *p* < 0.05, ** *p* < 0.01, *** *p* < 0.001, and **** *p* < 0.0001.

Overall, the explanted subcutaneous materials showed no evidence of infection or necrosis. There was an exudate fluid observed in the area of the native materials, which also shrank. Histopathology revealed inflammatory cell infiltration into the implanted materials (Figure 9). The inflammatory cell infiltration was significantly lower in the decellularized matrices compared with the native ones (Figure 9A,B). Lymphocytes, plasma cells, and macrophages were profusely observed in the native tissue implants. Fibroblasts could be clearly observed in the decellularized matrices. However, the newly formed blood vessels could only be detected in the decellularized material implants (Figure 9A,B).

## 4. Discussion

The utilization of biomimetic materials, such as autologous and allogeneic pericardia, appears to be an appropriate response to the clinical need for functional bio-replacements, which is rising quickly. The ECM structure that makes up pericardial tissues ensures pliability, conformability, and resistance to compression and promotes healing by repopulating cells. Moreover, they are not instantly resorbable [42]. Thus, they are ideal biological membranes for directed tissue regeneration. Patients’ comorbidities and the lack of donors represent important restrictions from an auto- or allotransplantation therapeutic perspective. Xenogeneic decellularized pericardia of animal origin have been shown to have characteristics that are comparable to those of human pericardia [31,46].

In the present study, a new approach to decellularizing xenogenic BP, PP, and PTV was introduced to develop biologically active and biocompatible biomaterials for applications in tissue engineering. The protocol merges the use of a biological enzymatic method (Trypsin) and a chemical method using non-ionic detergent (TX) and ionic detergent (SDS) (Trypsin + TX + SDS). Over the years, several decellularization approaches, including physical, chemical, and biological methods, have been employed to prepare decellularized ECMs for tissue engineering applications. The physical methods of decellularization comprise high hydrostatic pressure, freeze–thaw cycles, and superficial CO_2_ treatment [47]. The chemical protocols imply the use of ionic detergents such as sodium dodecyl sulfate (SDS) or non-ionic detergents such as Triton X-100 (TX) to destroy the cell membranes and separate their interior assembly. In addition to totally solubilizing cell and nucleic membranes and denaturing proteins, SDS is good at removing nuclear remnants. Meanwhile, TX, the detergent most often used in decellularization procedures, aims to break down lipid–lipid and lipid–protein associations while maintaining the integrity of protein–protein interaction [48,49,50]. Biological decellularization strategies usually utilize enzymes including Trypsin, dispase, and phospholipase A2. Furthermore, nucleases like DNAse are employed to encourage the fragmentation of leftover DNA into tiny pieces to maximize cell clearance and reduce immunological reactions [48].

In reconstructive or substitutive surgery, it has been shown that decellularized xenogeneic pericardia from other species [51,52], primarily bovine, provide comparable benefits and bypass the limitations of human heterologous tissues [38,53,54,55]. However, the number of comparative studies that have examined the impact of decellularization on various tissues from various species in depth is still limited. According to our knowledge, this work is the first to compare the effect of the Trypsin + TX + SDS protocol on the pericardia of bovine and porcine animals and porcine tunica vaginalis. This study reveals that the Trypsin + TX + SDS protocol is efficient at removing cells from pig pericardium and tunica vaginalis as well as bovine pericardium while having no negative effects on the ECMs of either species. The two species’ original discrepancy in tissue thickness still exists. This was confirmed by our findings from H & E staining and scanning electron microscopy. This is in line with [38,52]. In our study, decellularized bovine and porcine pericardia appeared to have the same capacity to tolerate tensile pressure as their native counterparts, which are distinct from porcine tunica vaginalis. A similar finding regarding bovine pericardium has been reported before [38,52]. However, the tensile strength of porcine pericardia was reported to decrease after TRICOL decellularization [52]. This finding confirms the subtle effect of the decellularization strategy adopted in the current investigation on the tensiometric properties of decellularized scaffolds. Moreover, these abilities appear to be inextricably linked to a species-specific biomechanical response of the tissues under study [52].

The eventual realization of the biological patches’ activities critically depends on their performance characteristics during the biodegradation process [56]. Since the degradation of biomaterials relies on the collagenase enzyme [57], in the present work, native and decellularized materials were stable in PBS. This confirmed the safety of preserving decellularized tissues in PBS for a long time till the time of utilization [16]. Any scaffold aiming to be a living substitute with the capacity for self-repair must offer a cell-friendly environment that is repopulation-permissive once implanted. Implanted scaffolds will frequently come into contact with mesenchymal cells in clinical settings, whether they are migrating from anastomotic tissues or being mobilized from the bone marrow (hBM-MSCs) [46,51,58]. DBP and DPP produce a high level of proliferation. This discovery holds promise for the maintenance of the key signals involved in the pericardium’s bioactivity and, consequently, for the ability to forecast the repopulation of decellularized scaffolds and the successful completion of a clinical application. It has been previously reported that despite several washing steps, it is not possible to eliminate traces of the surfactant from the treated material [59]. However, in our study, decellularized materials exhibited promising cytocompatibility. This is consistent with previous research on the tissue cytotoxicity of porcine pericardium decellularized with 0.1% SDS, which showed no cytotoxic effect on fibroblasts from human skin [60]. Likewise, in another study, bovine pericardia decellularized with low concentrations of SDS presented a low cytotoxic effect on hASCs [38]. On the other hand, in another investigation, SDS-treated tissues still exhibited a cytotoxic impact even after washing them for seven days, which was attributable to the significant anionic detergent potential [61]. Excessive detergent residue levels have been linked to decreased tensile strength and increased elastin fiber proteolysis, both of which may cause structural degeneration later on [62], which was not recorded in our study. The ultimate goal of tissue engineering is the creation of biological replacements that maintain, improve, or restore tissue function. In this field of research, growth factors, cells, and/or scaffolds are used to offer structural support and the potential for tissues to form following transplantation in the patient’s body. Despite the availability of a wide range of materials, there is rising interest in the necessity of biological tissue ECMs since they can transmit important cell signals during interactions with the host tissue [63]. Hence, via cultivation with r-AdMSCs, decellularized materials’ cytocompatibility was evaluated. Research has revealed that cell culture on the pericardium and tunica vaginalis can be problematic since the tissue may have small pores and insufficient area for cell growth [64,65]. On the other hand, our findings show that the decellularized BP, PP, and PTV could still maintain the cells after 30 days of culture. Also, we found cells deep within the tissue, indicating migration through the ECM of the tissue, which is consistent with previous studies [38,66].

In the present study, the biocompatibility and biodegradability of the materials were assessed after subcutaneous implantation using H & E staining. Our findings reveal that the inflammatory cell infiltration was severe in the native tissues. On the other hand, decellularized materials did not induce potent inflammation. Similar findings were reported by [44], who attributed this to the faint amount of residual DNA after decellularization. Fibroblasts and newly formed blood vessels could be clearly detected in the DBP, DPP, and DPTV implants in our experiment. These results confirm the biocompatibility of these materials. Moreover, the decellularization protocol increases the pore size of tissues [44]. The presence of a few macrophages in decellularized matrices implies their biodegradability [67]. Ideal biomedical materials for implantation surgeries should exhibit properties like strength, biocompatibility, biodegradability, availability of sterile conditions, affordability, and sustainability [46,51]. All these characteristics are present in Trypsin + TX + SDS-decellularized bovine pericardia and porcine pericardia and tunica vaginalis, which are produced using an affordable decellularization method. They can also be given to or provided by tissue banks in the best possible sterilized and conserved condition for their off-the-shelf dispersion in clinical procedures [68,69]. Regarding surgical applications, the decellularized porcine pericardium’s inferior thickness is particularly desirable for cardiovascular applications like the engineering of cardiac tissue and the construction of percutaneous devices, such as transcatheter heart valve replacements [42]. On the other hand, decellularized bovine pericardia and porcine tunica vaginalis might be expected to show satisfactory tensile strength, failure strain, and thickness for applications such as gastrointestinal reconstructive settings like repairing abdominal wall defects.

## 5. Limitations of the Present Study

Despite the findings of in vitro cytocompatibility, corresponding H & E staining, and in vivo S/C implantation, confirming the biocompatibility of different decellularized biomaterials, in the present study, the proliferation assay is essential to precisely quantify the degree of proliferation among these different materials. Moreover, the implementation of bioinformatic techniques like proteomics in future experiments could be helpful to elaborate in more detail on the influence of the decellularization protocol on the fine structure of decellularized matrices and confirm the complete removal of any antigenic components that may induce the immune system.

## 6. Conclusions

In conclusion, Trypsin + TX + SDS is a promising novel protocol for the successful preparation of efficient decellularized extracellular matrices with high potential for tissue engineering applications. Acellular pericardia and tunica vaginalis scaffolds offered comparable profiles in terms of structural, biomechanical, and bioactivity properties after investigation. They may potentially be considered auspicious, bioactive materials for reconstruction and replacement purposes. Also, the comparison provided here showed that the less clinically employed acellular porcine pericardium could be a biomaterial of interest for use in surgical reconstruction or replacement. On the other hand, decellularized bovine pericardia and porcine tunica vaginalis are more appropriate for reconstruction of tension-bearing organs.

## Data Availability

The original data used in the study are stated in the article; further inquiries can be directed to the corresponding authors.

## References

[1] Levitt M. (2015). Could the organ shortage ever be met?. Life Sci. Soc. Policy.

[2] El-Husseiny H.M., Mady E.A., Hamabe L., Abugomaa A., Shimada K., Yoshida T., Tanaka T., Yokoi A., Elbadawy M., Tanaka R. (2022). Smart/stimuli-responsive hydrogels: Cutting-edge platforms for tissue engineering and other biomedical applications. Mater. Today Bio.

[3] El-Husseiny H.M., Mady E.A., El-Dakroury W.A., Doghish A.S., Tanaka R. (2022). Stimuli-Responsive Hydrogels: Smart State of-the-Art Platforms for Cardiac Tissue Engineering. https://www.researchsquare.com/article/rs-2011475/v1.

[4] El-Husseiny H.M., Mady E.A., El-Dakroury W.A., Zewail M.B., Noshy M., Abdelfatah A.M., Doghish A.S. (2022). Smart/stimuli-responsive hydrogels: State-of-the-art platforms for bone tissue engineering. Appl. Mater. Today.

[5] El-Husseiny H.M., Mady E.A., Radwan Y., Nagy M., Abugomaa A., Elbadawy M., Tanaka R., Ali G.A.M., Makhlouf A.S.H. (2022). Hybrid Biodegradable Polymeric Scaffolds for Cardiac Tissue Engineering. Handbook of Biodegradable Materials.

[6] Abd Elkodous M., El-Husseiny H.M., El-Sayyad G.S., Hashem A.H., Doghish A.S., Elfadil D., Radwan Y., El-Zeiny H.M., Bedair H., Ikhdair O.A. (2021). Recent advances in waste-recycled nanomaterials for biomedical applications: Waste-to-wealth. Nanotechnol. Rev..

[7] Hunsberger J., Neubert J., Wertheim J.A., Allickson J., Atala A. (2016). Bioengineering Priorities on a Path to Ending Organ Shortage. Curr. Stem Cell Rep..

[8] Doghish A.S., El-Husseiny A.A., Abdelmaksoud N.M., El-Mahdy H.A., Elsakka E.G.E., Abdel Mageed S.S., Mahmoud A.M.A., Raouf A.A., Elballal M.S., El-Dakroury W.A. (2023). The interplay of signaling pathways and miRNAs in the pathogenesis and targeted therapy of esophageal cancer. Pathol.—Res. Pract..

[9] Doghish A.S., Ismail A., El-Mahdy H.A., Elkhawaga S.Y., Elsakka E.G.E., Mady E.A., Elrebehy M.A., Khalil M.A.F., El-Husseiny H.M. (2023). miRNAs insights into rheumatoid arthritis: Favorable and detrimental aspects of key performers. Life Sci..

[10] El-Mahdy H.A., Mohamadin A.M., Abulsoud A.I., Khidr E.G., El-Husseiny A.A., Ismail A., Elsakka E.G.E., Mokhlis H.A., El-Husseiny H.M., Doghish A.S. (2023). miRNAs as potential game-changers in head and neck cancer: Future clinical and medicinal uses. Pathol.—Res. Pract..

[11] Mady E.A., Doghish A.S., El-Dakroury W.A., Elkhawaga S.Y., Ismail A., El-Mahdy H.A., Elsakka E.G.E., El-Husseiny H.M. (2023). Impact of the mother’s gut microbiota on infant microbiome and brain development. Neurosci. Biobehav. Rev..

[12] Perkel J.M. (2016). Xenotransplantation makes a comeback. Nat. Biotechnol..

[13] Mohiuddin M.M., Singh A.K., Corcoran P.C., Hoyt R.F., Thomas M.L., Ayares D., Horvath K.A. (2014). Genetically engineered pigs and target-specific immunomodulation provide significant graft survival and hope for clinical cardiac xenotransplantation. J. Thorac. Cardiovasc. Surg..

[14] El-Husseiny H. (2017). Evaluation of Some Prosthetic Implants for Surgical Management of Different Varieties of Hernias in Domestic Animals.

[15] El-Husseiny M. (2019). Platelet rich fibrin augmented versus non-augmented glycerolized bovine pericardium and polypropylene mesh for repairing of large abdominal wall defects. Eur. J. Med. Nat. Sci..

[16] Ramm R., Goecke T., Theodoridis K., Hoeffler K., Sarikouch S., Findeisen K., Ciubotaru A., Cebotari S., Tudorache I., Haverich A. (2020). Decellularization combined with enzymatic removal of N-linked glycans and residual DNA reduces inflammatory response and improves performance of porcine xenogeneic pulmonary heart valves in an ovine in vivo model. Xenotransplantation.

[17] Gilbert T.W., Sellaro T.L., Badylak S.F. (2006). Decellularization of tissues and organs. Biomaterials.

[18] Crapo P.M., Gilbert T.W., Badylak S.F. (2011). An overview of tissue and whole organ decellularization processes. Biomaterials.

[19] Parmaksiz M., Dogan A., Odabas S., Elçin A.E., Elçin Y.M. (2016). Clinical applications of decellularized extracellular matrices for tissue engineering and regenerative medicine. Biomed. Mater..

[20] Vorotnikova E., McIntosh D., Dewilde A., Zhang J., Reing J.E., Zhang L., Cordero K., Bedelbaeva K., Gourevitch D., Heber-Katz E. (2010). Extracellular matrix-derived products modulate endothelial and progenitor cell migration and proliferation in vitro and stimulate regenerative healing in vivo. Matrix Biol..

[21] Xu C.C., Chan R.W., Weinberger D.G., Efune G., Pawlowski K.S. (2010). A bovine acellular scaffold for vocal fold reconstruction in a rat model. J. Biomed. Mater. Res. Part A.

[22] El-Husseiny H.M., Kaneda M., Mady E.A., Yoshida T., Doghish A.S., Tanaka R. (2023). Impact of Adipose Tissue Depot Harvesting Site on the Multilineage Induction Capacity of Male Rat Adipose-Derived Mesenchymal Stem Cells: An In Vitro Study. Int. J. Mol. Sci..

[23] El-Husseiny H.M., Mady E.A., Helal M.A., Tanaka R. (2022). The Pivotal Role of Stem Cells in Veterinary Regenerative Medicine and Tissue Engineering. Vet. Sci..

[24] Hendawy H., Uemura A., Ma D., Namiki R., Samir H., Ahmed M.F., Elfadadny A., El-Husseiny H.M., Chieh-Jen C., Tanaka R. (2021). Tissue Harvesting Site Effect on the Canine Adipose Stromal Vascular Fraction Quantity and Quality. Animals.

[25] Sharun K., Chandran D., Manjusha K.M., Mankuzhy P.D., Kumar R., Pawde A.M., Dhama K., El-Husseiny H.M. (2023). Amarpal. Advances and prospects of platelet-rich plasma therapy in veterinary ophthalmology. Vet. Res. Commun..

[26] Gilbert T.W., Freund J.M., Badylak S.F. (2009). Quantification of DNA in Biologic Scaffold Materials. J. Surg. Res..

[27] Aguiari P., Iop L., Favaretto F., Fidalgo C.M.L., Naso F., Milan G., Vindigni V., Spina M., Bassetto F., Bagno A. (2017). In vitro comparative assessment of decellularized bovine pericardial patches and commercial bioprosthetic heart valves. Biomed. Mater..

[28] Keane T.J., Londono R., Turner N.J., Badylak S.F. (2012). Consequences of ineffective decellularization of biologic scaffolds on the host response. Biomaterials.

[29] McDade J.K., Brennan-Pierce E.P., Ariganello M.B., Labow R.S., Michael Lee J. (2013). Interactions of U937 macrophage-like cells with decellularized pericardial matrix materials: Influence of crosslinking treatment. Acta Biomater..

[30] Gauvin R., Marinov G., Mehri Y., Klein J., Li B., Larouche D., Guzman R., Zhang Z., Germain L., Guidoin R. (2013). A comparative study of bovine and porcine pericardium to highlight their potential advantages to manufacture percutaneous cardiovascular implants. J. Biomater. Appl..

[31] Manji R.A., Zhu L.F., Nijjar N.K., Rayner D.C., Korbutt G.S., Churchill T.A., Rajotte R.V., Koshal A., Ross D.B. (2006). Glutaraldehyde-Fixed Bioprosthetic Heart Valve Conduits Calcify and Fail From Xenograft Rejection. Circulation.

[32] Schoen F.J., Levy R.J. (2005). Calcification of Tissue Heart Valve Substitutes: Progress Toward Understanding and Prevention. Ann. Thorac. Surg..

[33] Mendoza-Novelo B., Avila E.E., Cauich-Rodríguez J.V., Jorge-Herrero E., Rojo F.J., Guinea G.V., Mata-Mata J.L. (2011). Decellularization of pericardial tissue and its impact on tensile viscoelasticity and glycosaminoglycan content. Acta Biomater.

[34] Fentie I.H., Allen D.J., Schenck M.H., Didio L.J. (1986). Comparative electron microscopic study of bovine, porcine and human parietal pericardium, as materials for cardiac valve bioprostheses. J. Submicrosc. Cytol..

[35] Huen K.H., Macaraeg A., Davis-Dao C.A., Williamson S.H., Boswell T.C., Chuang K.-w., Stephany H.A., Wehbi E.J., Khoury A.E. (2022). Single-Layer Acellular Porcine Bladder Matrix as Graft in Corporoplasty for Ventral Curvature in Pediatric Proximal Hypospadias Repair: An Initial Experience. Urology.

[36] Huen K.H., Macaraeg A., Khoury A.E. (2022). Ventral penile lengthening using tunica vaginalis flap for correction of curvature in proximal hypospadias repair: Technical aspects. Urol. Video J..

[37] Bružauskaitė I., Bironaitė D., Bagdonas E., Bernotienė E. (2016). Scaffolds and cells for tissue regeneration: Different scaffold pore sizes—Different cell effects. Cytotechnology.

[38] Heuschkel M.A., Leitolis A., Roderjan J.G., Suss P.H., Luzia C.A.O., da Costa F.D.A., Correa A., Stimamiglio M.A. (2019). In vitro evaluation of bovine pericardium after a soft decellularization approach for use in tissue engineering. Xenotransplantation.

[39] Mallis P., Michalopoulos E., Dimitriou C., Kostomitsopoulos N., Stavropoulos-Giokas C. (2017). Histological and biomechanical characterization of decellularized porcine pericardium as a potential scaffold for tissue engineering applications. Bio-Med. Mater. Eng..

[40] Chantawong P., Tanaka T., Uemura A., Shimada K., Higuchi A., Tajiri H., Sakura K., Murakami T., Nakazawa Y., Tanaka R. (2017). Silk fibroin-Pellethane^®^ cardiovascular patches: Effect of silk fibroin concentration on vascular remodeling in rat model. J. Mater. Sci. Mater. Med..

[41] Koyanagi E., Tara S., Sakata C., Shimada K., Kato K., Miyachi H., Tanaka R., Nakazawa Y. (2022). A novel gradient and multilayered sheet with a silk fibroin/polyvinyl alcohol core–shell structure for bioabsorbable arterial grafts. J. Biomed. Mater. Res. Part A.

[42] Choe J.A., Jana S., Tefft B.J., Hennessy R.S., Go J., Morse D., Lerman A., Young M.D. (2018). Biomaterial characterization of off-the-shelf decellularized porcine pericardial tissue for use in prosthetic valvular applications. J. Tissue Eng. Regen. Med..

[43] Soares L.G., de Oliveira F.S., Queiroz A., de Medeiros A., Bariani Junior A.F., Fechis A.D.S., Rocha T. (2021). Biomechanics of the fresh and conserved bovine pericardium. Anat. Histol. Embryol..

[44] Alizadeh M., Rezakhani L., Khodaei M., Soleimannejad M., Alizadeh A. (2021). Evaluating the effects of vacuum on the microstructure and biocompatibility of bovine decellularized pericardium. J. Tissue Eng. Regen. Med..

[45] Ni X., Shan X., Xu L., Yu W., Zhang M., Lei C., Xu N., Lin J., Wang B. (2021). Adipose-derived stem cells combined with platelet-rich plasma enhance wound healing in a rat model of full-thickness skin defects. Stem Cell Res. Ther..

[46] Iop L., Palmosi T., Dal Sasso E., Gerosa G. (2018). Bioengineered tissue solutions for repair, correction and reconstruction in cardiovascular surgery. J. Thorac. Dis..

[47] Topuz B., Günal G., Guler S., Aydin H.M., Caballero D., Kundu S.C., Reis R.L. (2020). Chapter 4—Use of supercritical CO2 in soft tissue decellularization. Methods in Cell Biology.

[48] Fernández-Pérez J., Ahearne M. (2019). The impact of decellularization methods on extracellular matrix derived hydrogels. Sci. Rep..

[49] Mendibil U., Ruiz-Hernandez R., Retegi-Carrion S., Garcia-Urquia N., Olalde-Graells B., Abarrategi A. (2020). Tissue-Specific Decellularization Methods: Rationale and Strategies to Achieve Regenerative Compounds. Int. J. Mol. Sci..

[50] White L.J., Taylor A.J., Faulk D.M., Keane T.J., Saldin L.T., Reing J.E., Swinehart I.T., Turner N.J., Ratner B.D., Badylak S.F. (2017). The impact of detergents on the tissue decellularization process: A ToF-SIMS study. Acta Biomater..

[51] Filippi R., Schwarz M., Voth D., Reisch R., Grunert P., Perneczky A. (2001). Bovine pericardium for duraplasty: Clinical results in 32 patients. Neurosurg. Rev..

[52] Zouhair S., Dal Sasso E., Tuladhar S.R., Fidalgo C., Vedovelli L., Filippi A., Borile G., Bagno A., Marchesan M., De Rossi G. (2020). A Comprehensive Comparison of Bovine and Porcine Decellularized Pericardia: New Insights for Surgical Applications. Biomolecules.

[53] Bel A., Kachatryan L., Bruneval P., Peyrard S., Gagnieu C., Fabiani J.-N., Menasché P. (2010). A new absorbable collagen membrane to reduce adhesions in cardiac surgery. Interact. CardioVascular Thorac. Surg..

[54] Gubitosi A., Docimo G., Parmeggiani D., Pirozzi R., Vitiello C., Schettino P., Avellino M., Casalino G., Amato M., Ruggiero R. (2014). Acellular bovine pericardium dermal matrix in immediate breast reconstruction after Skin Sparing Mastectomy. Int. J. Surg..

[55] Le B., Borzabadi-Farahani A., Nielsen B. (2016). Treatment of Labial Soft Tissue Recession Around Dental Implants in the Esthetic Zone Using Guided Bone Regeneration With Mineralized Allograft: A Retrospective Clinical Case Series. J. Oral Maxillofac. Surg..

[56] Cao G., Wang C., Fan Y., Li X. (2020). Biomimetic SIS-based biocomposites with improved biodegradability, antibacterial activity and angiogenesis for abdominal wall repair. Mater. Sci. Eng. C.

[57] Cheng S., Liu X., Qian Y., Maitusong M., Yu K., Cao N., Fang J., Liu F., Chen J., Xu D. (2022). Double-Network Hydrogel Armored Decellularized Porcine Pericardium as Durable Bioprosthetic Heart Valves. Adv. Healthc. Mater..

[58] Williams D.F. (2019). Challenges With the Development of Biomaterials for Sustainable Tissue Engineering. Front. Bioeng. Biotechnol..

[59] Spina M., Naso F., Zancan I., Iop L., Dettin M., Gerosa G. (2014). Biocompatibility issues of next generation decellularized bioprosthetic devices. Conference Papers in Science.

[60] Tran H.L.B., Dinh T.T.H., Nguyen M.T.N., To Q.M., Pham A.T.T. (2016). Preparation and characterization of acellular porcine pericardium for cardiovascular surgery. Turk. J. Biol..

[61] Liu Z.Z., Wong M.L., Griffiths L.G. (2016). Effect of bovine pericardial extracellular matrix scaffold niche on seeded human mesenchymal stem cell function. Sci. Rep..

[62] Kagan H.M., Crombie G.D., Jordan R.E., Lewis W., Franzblau C. (1972). Proteolysis of elastin-ligand complexes. Stimulation of elastase digestion of insoluble elastin by sodium dodecyl sulfate. Biochemistry.

[63] Lakshmanan R., Krishnan U.M., Sethuraman S. (2012). Living cardiac patch: The elixir for cardiac regeneration. Expert Opin. Biol. Ther..

[64] Wei H.J., Chen S.C., Chang Y., Hwang S.M., Lin W.W., Lai P.H., Chiang H.K., Hsu L.F., Yang H.H., Sung H.W. (2006). Porous acellular bovine pericardia seeded with mesenchymal stem cells as a patch to repair a myocardial defect in a syngeneic rat model. Biomaterials.

[65] Hollister S.J. (2005). Porous scaffold design for tissue engineering. Nat. Mater..

[66] Lepedda A.J., Nieddu G., Formato M., Baker M.B., Fernández-Pérez J., Moroni L. (2021). Glycosaminoglycans: From Vascular Physiology to Tissue Engineering Applications. Front. Chem..

[67] Kim H.D., Hong X., An Y.-H., Park M.J., Kim D.-G., Greene A.K., Padwa B.L., Hwang N.S., Lin R.-Z., Melero-Martin J.M. (2021). A Biphasic Osteovascular Biomimetic Scaffold for Rapid and Self-Sustained Endochondral Ossification. Adv. Healthc. Mater..

[68] Zouhair S., Aguiari P., Iop L., Vásquez-Rivera A., Filippi A., Romanato F., Korossis S., Wolkers W.F., Gerosa G. (2019). Preservation strategies for decellularized pericardial scaffolds for off-the-shelf availability. Acta Biomater..

[69] Fidalgo C., Iop L., Sciro M., Harder M., Mavrilas D., Korossis S., Bagno A., Palù G., Aguiari P., Gerosa G. (2018). A sterilization method for decellularized xenogeneic cardiovascular scaffolds. Acta Biomater..

